# Challenges and Opportunities in Border Health^1^

**Published:** 2004-12-15

**Authors:** Joel Rodríguez-Saldaña

Approximately 11.5 million people reside in the 42 counties and 39 Mexican municipalities located along the U.S.-Mexico border, and 86% of those people reside in 14 pairs of sister cities, metropolitan areas divided by the international border (1). Border residents share similar resources and environmental problems: issues of great concern include air quality, water quantity and quality, and animal control. The communities along the border are economically and socially interdependent, with more than 1 million legal northbound crossings every day. The need to establish cooperation between the United States and Mexico for improving health has led to collaborative initiatives between the public and private sectors (1). The principal health problems at the U.S.-Mexico border are characterized by disparities in health systems (2), which result from the lower health standards and socioeconomic conditions of Mexican border communities compared with U.S. border communities.

Health-system disparities produce differences in and barriers to health care access and use(3,4). Documented cases that demand the creation of programs across the U.S.-Mexico border show different rates in the prevalence of infectious disease, including hepatitis A, salmonella, tuberculosis, dengue fever, and *Helicobacter pylori* infection (5,6). The magnitude and relevance of infectious disease as a major concern along the U.S.-Mexico border have prompted the establishment of binational agreements, such as the U.S.-Mexico Border Infectious Disease Surveillance Project, with the purpose of enhancing the effectiveness of infectious disease prevention (7). On the other hand, populations on both sides of the border share the impact of diseases — such as obesity and diabetes — resulting from similar lifestyle changes. The prevalence rate for diabetes along the U.S.-Mexico border is nearly 50% higher than the rate for the rest of the United States, and Hispanics are more vulnerable to suffering the burden of chronic complications because of genetic, economic, social, behavioral, and psychological factors.

This issue of *Preventing Chronic Disease* includes an introduction and overview (8) as well as additional articles on the Border Health Strategic Initiative (*Border Health ¡SI!*), a comprehensive community approach to diabetes prevention and control primarily concentrated in Yuma and Santa Cruz counties in Arizona. *Border Health ¡SI!* is based on models of community capacity building and community change and was established through a partnership between several border community groups and the University of Arizona. In addition to being comprehensive and community oriented, *Border Health ¡SI!* was designed to be acceptable to stakeholders, effective in fostering and sustaining change, adaptable to other communities, sustainable after funding, and process and outcome focused.

To reduce the incidence of diabetes among individuals with impaired glucose tolerance, *Border Health ¡SI!* has emphasized the management of risk factors such as obesity through lifestyle changes (e.g., nutritional counseling, increased physical activity, modest weight loss). The program has also focused on community-based diabetes care provided by a multidisciplinary team that targets patients with diabetes, their families, and their health care providers. Community-health outreach workers called *promotores de salud* have been instrumental in implementing interventions designed to change personal health risk factors.

The introductory article also describes the formation of community-based coalitions called Special Action Groups (SAGs), whose primary goal is to identify and implement plans for policy and environmental change. Meister et al (9) provide details on how the SAGs in two communities were formed and how they promoted activities to support physical activity and nutrition, and Steinfelt (10) reports on her experience as the community coordinator responsible for orchestrating SAG activities. Other articles in this issue, described below, provide examples of target populations.

Ingram et al report on the effectiveness of a series of diabetes education classes to assist participants in gaining knowledge and skills necessary to be physically active, control diet, monitor blood sugar, take medications, and be aware of complications (11). *Promotores de salud* play a key role in conducting outreach, participating in patient education, and providing educational support in an overall framework in which individual ability to manage diabetes is not separated from community context and support for diabetes care. Community health centers administered the program and provided a coordinator. Academic partners provided technical assistance and conducted evaluations. The culturally competent curriculum employed a variety of teaching methods to educate participants on how diabetes affects the body. In addition, program staff measured blood glucose, weight, and blood pressure at each of five weekly classes. Improvements in self-management behaviors, HbA1c, random blood glucose, and blood pressure were documented after five weeks. The authors conclude that successful implementation of a program like *Border Health ¡SI!* includes five essential elements: basic diabetes education, peer outreach and support, integration of diabetes and clinical care, access to medical care and medication, and sustainability.

Teufel-Shone et al (12) describe how the University of Arizona and two community health agencies collaborated to design, pilot, and assess the feasibility of a lay health-outreach, worker-delivered diabetes education program for families. The culturally appropriate program addressed family food choices, physical activity, behavior change, communication, and support behaviors. Seventy-two families participated, and pre- and post-evaluations showed an increase in knowledge of diabetes risk factors and an increase in family efficacy to change food and activity behaviors.

Staten et al report their findings after implementing the School Health Index (SHI) in 13 schools in two counties along the U.S.-Mexico border as part of *Border Health ¡SI! *between 2000 and 2003 (13). The alarming increase in childhood obesity is a contributing factor to the escalating rate of type 2 diabetes among adolescents. Although the school environment is shown to neglect promotion of physical activity (e.g., by eliminating or not offering physical education classes) and good nutrition (e.g., by selling candy in vending machines), it offers opportunities to combat obesity and diabetes. The SHI is a team-based program launched by the Centers for Disease Control and Prevention in 2000 as a self-assessment and planning tool for health promotion. The SHI enables schools to identify strengths and weaknesses of physical activity and nutrition policies and programs and to develop action plans for improving student health. *Border Health ¡SI! *supported the hiring and training of an external (i.e., not part of the school system) SHI coordinator in each county who worked with the schools to implement the SHI, develop action plans, and monitor progress. Process and participation varied from school to school, but most schools made at least one immediate change in the school environment to promote student health. Analysis of short-term and intermediate outcomes of the SHI at these schools will be of great additional value.

Staten et al also report on *Pasos Adelante*, a curriculum designed in cultural context aimed at preventing diabetes, cardiovascular disease, and other chronic diseases in Hispanic populations (14). The 12-week program was facilitated by *promotoras de salud *in two counties along the Arizona-Sonora, Mexico border. Sessions included physical activity. Walking clubs were established that could continue after the program concluded. Approximately 250 people participated in *Pasos Adelante. *Analysis of pre- and post-program questionnaires demonstrated a significant increase in moderate to vigorous walking among participants as well as positive changes in nutritional patterns. The success of the *Pasos Adelante* curriculum shows that a culturally appropriate educational program can motivate people in border communities to adopt healthier lifestyle behaviors.

In a related article on original research, Abarca et al (15) illustrate how community indicators were used to assess nutrition in communities targeted by *Border Health ¡SI!*. Local grocery store purchases were selected as an indicator, and a structured 26-question interview was developed and administered to grocery store managers. In addition, the investigators gathered data from milk distributors serving these communities. Results showed that food items with a higher fat and higher caloric content were favored. The authors suggest that barriers to acceptance of healthier food items include lack of knowledge concerning healthy foods and their prices. They conclude that more interventions are needed to change dietary patterns, improve overall health, and prevent and control diabetes in these communities.

Schachter et al report their findings on implementing national diabetes guidelines in five border-community health centers (two in Arizona and three in Texas) (16). Each center selected their top four or five indicators of diabetes care and performed baseline audits of medical records in a minimal sample of 12 to 15 charts. Percentage level of compliance for each indicator was compared with the average percentage level of overall diabetes care compliance for each community health center. Priorities varied from clinic to clinic, but the majority of indicators showed improvement. All participating centers expressed interest in improving performance. Only three centers, however, were audited again 24 months later: two maintained or increased improvements, and one lost ground. As reported in other studies(17), translating guidelines into practice is easier said than done: "Between the health care we have and the care we could have lies not just a gap, but a chasm"(18).

Although there is increasing evidence of improvements in diabetes care, not all people with diabetes are experiencing these benefits(19). Addressing the complexities of diabetes management, improving the established systems of care, and recognizing the decisive role of personal, social, and economic factors on diabetes care for each individual with diabetes are the greatest health challenges of our time. The U.S.-Mexico border is a unique example of the interaction of global interdependence: the challenges of providing formal diabetes education in border communities are overwhelming (11). It would be desirable for this interdependence to produce better standards of living and health for all, but evidence confirms that this is not the case(1). The Border Health Strategic Initiative is an illustrative example of a long and successful record of collaborative work, with defined goals, including process and outcome analysis. Resolution of the many challenges that the emerging epidemic rates of diabetes presents at the U.S.-Mexico border will certainly apply to other scenarios of health disparity.
